# Tendon Remodeling in Response to Resistance Training, Anabolic Androgenic Steroids and Aging

**DOI:** 10.3390/cells7120251

**Published:** 2018-12-07

**Authors:** Vinicius Guzzoni, Heloisa Sobreiro Selistre-de-Araújo, Rita de Cássia Marqueti

**Affiliations:** 1 Departamento de Biologia Molecular e Celular, Universidade Federal da Paraíba, João Pessoa 58051-970, Paraíba, Brazil; 2 Department of Physiological Sciences, Federal University of São Carlos, São Carlos 13565-205, São Paulo, Brazil; hsaraujo@ufscar.br; 3 Graduate Program of Rehabilitation Science, University of Brasilia, Distrito Federal, Brasília 70840-901, Distrito Federal, Brazil; marqueti@gmail.com

**Keywords:** tendon, resistance training, anabolic androgenic steroids, aging, extracellular matrix

## Abstract

Exercise training (ET), anabolic androgenic steroids (AAS), and aging are potential factors that affect tendon homeostasis, particularly extracellular matrix (ECM) remodeling. The goal of this review is to aggregate findings regarding the effects of resistance training (RT), AAS, and aging on tendon homeostasis. Data were gathered from our studies regarding the impact of RT, AAS, and aging on the calcaneal tendon (CT) of rats. We demonstrated a series of detrimental effects of AAS and aging on functional and biomechanical parameters, including the volume density of blood vessel cells, adipose tissue cells, tendon calcification, collagen content, the regulation of the major proteins related to the metabolic/development processes of tendons, and ECM remodeling. Conversely, RT seems to mitigate age-related tendon dysfunction. Our results suggest that AAS combined with high-intensity RT exert harmful effects on ECM remodeling, and also instigate molecular and biomechanical adaptations in the CT. Moreover, we provide further information regarding the harmful effects of AAS on tendons at a transcriptional level, and demonstrate the beneficial effects of RT against the age-induced tendon adaptations of rats. Our studies might contribute in terms of clinical approaches in favor of the benefits of ET against tendinopathy conditions, and provide a warning on the harmful effects of the misuse of AAS on tendon development.

## 1. Introduction

The calcaneal tendon (CT), known as the Achilles tendon, is more susceptible to tendinopathy since it is subjected to greater mechanical loading [1,2,3,4]. Aging has been shown to induce detrimental effects on tendons [2,5], while exercise training (ET) seems to mitigate these age-induced detrimental effects. In addition, anabolic-androgenic steroids (AAS) have been shown to evoke potential effects on skeletal muscle [6,7,8,9,10], suggesting that tendons might be affected by AAS misuse [11,12,13,14]. It has also been demonstrated that aging can cause detrimental effects in tendon composition, however mechanical loading may improve tendon structure and content and diminish the detrimental effects of AAS and aging [15,16]. Thus, the purpose of this narrative review is to provide an in-depth understanding of the major effects of ET, particularly resistance training (RT), AAS and aging on tendon remodeling of rats. Data were gathered from our studies regarding the impact of RT, AAS and aging on CT adaptations. Nevertheless, the evidence of tendon remodeling in response to resistance training, AAS and aging remain inconclusive, further highlighting the importance of this review.

## 2. Structure and Function of Tendons

**Figure 1:**
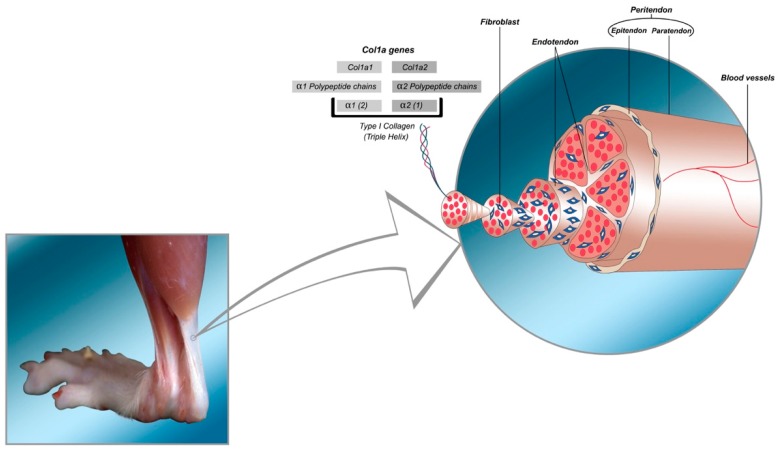
Structure of tendon. The tendon is composed of type I collagen fibers. Type I collagen is the major structural component of the tendon. Col1a1 and Col1a2 code for collagen α1(I) and α2(I) polypeptides, respectively. Type I collagen triple-helical molecules containing two α1(I) and one α2(I) chains assemble into fibrils that combine to form fibers. Tendon fibroblasts reside between collagen fibers. Fibers are surrounded by a connective tissue, the endotendon, which also contains fibroblasts. Fibers combine to form fascicules. Tendons are ensheathed by an outer layer of connective tissue (epitendon), which is surrounded by another layer of connective tissue (paratendon). Together, the epitendon and paratendon external sheaths constitute the peritendon. Adapted from Nourissat et al. 2015 [30] and Lipman et al. 2018 [56].

Tendons are soft tissues considered as inert and inextensible structures, even though they confer elastic properties that influence the muscle-tendon unit function [17,18]. Tendons constitute important structures of the musculoskeletal system that transmit muscle-generating tensile force to bones, resulting in movement [19,20,21,22]. While tendons are attached to muscles through the myotendinous junction, tendons are connected to bone through a fibrocartilaginous tissue called enthesis [23]. The force transmission from muscles to bones is possible because of the complex internal architecture of the tendon [24]. The structure of the tendon is arranged hierarchically by the tropocollagen, collagen fibrils, fibers, and fascicles [25] (Figure 1). A fascicle is a bundle of fibers [25]. Soluble tropocollagen molecules form cross-links to generate insoluble collagen, whose molecules are gradually assembled to arrange collagen fibrils [24]. A group of collagen fibrils, in turn, gives rise to collagen fiber, which corresponds to the basic unit of the tendon [21,24]. The fibrils are oriented within one collagen fiber in a three-dimensional arrangement (longitudinally, horizontally, and transversely), which forms spiral-type plait groups [24,26]. Sheaths of connective tissue surround the collagen fibers (endotendon) and the entire tendon (epitendon) [24]. While the endotendon is a thin reticular network of connective tissue inside the tendon, the epitendon constitutes a dense fibrillar network of collagen [26,27]. The endotendon arrangement enables the fiber groups to glide on each other and drives blood vessels and nerves to a deeper portion of the tendon [28].

Tendon cells (tenoblasts, tenocytes, chondrocytes, synovial cells, and vascular cells) are located between fibril chains and synthesize proteins of the extracellular matrix (ECM), constituted primarily by collagens, large proteoglycans, and small leucine-rich proteoglycans [21]. Type I collagen, elastin, and ECM elements are synthesized by tenocytes, which are fibroblast-like cells situated within collagen fibers and the surrounding endotenon [29,30]. Numerous ECM molecules, including collagens, elastin, proteoglycans, and glycoproteins are involved in the fibrillogenesis of type I collagen [31]. The number and diameter of collagen fibers vary in different tendons [24,32,33]. Collagen fibers and fibrils in tendons present a wavy configuration in the resting state, their shape modifying when the tendon is stretched [27]. A periodic waveform configuration is observed in collagen fibers [34], known as crimp morphology [35,36,37,38]. Moreover, collagen fibers are assembled in parallel bundles and aligned along the long axis of the tendon. This arrangement favors the tissue to better respond to mechanical loading [37].

Elastic fibers are sparsely distributed among tendons and account for approximately 1–2% of the dry mass of the tendon [24,39,40]. Elastic fibers are made of fibrillins (fibrillins 1 and 2) and elastin [41], which contribute collectively to the structural integrity and recovery of the wavy configuration of the collagen fibers after stretching [24,42]. As a crucial component in ECM, elastin ensures elastic stretching and recoiling of tissue, cooperating with collagen for tensile resistance [43] and regulating the interactions between cells and the extracellular matrix [44].

The ground substance of tendons consists of proteoglycans (PGs), glycosaminoglycans (GAGs), structural glycoproteins, and water, surrounding the collagen [24]. PGs are core proteins where one or more GAGs are covalently attached [24]. They enable rapid diffusion of water-soluble molecules and migration of cells [24]. The water-binding capacity of proteoglycans and GAGs is essential for stabilization of collagen fibrillogenesis, maintenance of ionic homeostasis, and elasticity of a tendon against shear and compressive forces [24]. Proteoglycans function either as lubricators or organizers of collagen fibril assembly [24,45], retaining water and conferring improved elasticity to tendons [4,24]. Decorin accounts for roughly 80% of the total proteoglycan content in the tissue [46,47]. Laminin, in turn, is found in both the vascular walls and myotendinous junction of tendons [26,48]. Inorganic components correspond to less than 0.2% of the tendon dry mass, including calcium, magnesium, manganese, cadmium, cobalt, copper, zinc, lithium, nickel, fluoride, phosphor, and silicon. These elements play an important role in the growth, development, and metabolism of the tissue. Copper, manganese, and calcium ions seem to assist in the formation of collagen cross-linking and enzymatic reactions related to synthesis of connective tissue molecules [49]. Tendon cells are also involved in energy metabolism, given that tendon cells contain enzymes related to the aerobic Krebs cycle, anaerobic glycolysis, and pentose phosphate shunt [24,50]. In fact, high metabolic activity and intense synthesis of the matrix components has been observed in young tenoblasts [24]. Moreover, physical activity affects human tendons directly through increased metabolic activity [18,51,52] and elevated collagen synthesis [53].

A new type of tendon cells, tendon stem cells (TSPCs) was recently discovered to be present in tendons and ligaments [21]. TSPCs differ from resident tenocytes in shape configuration, proliferation potential, and stem cell specific marker expression [4]. Tendon cell lines express genes of adipogenic, osteogenic, and chondrogenic differentiation pathways, suggesting their capacity to differentiate in vitro [54,55].

## 3. Effect of Training Modalities on Tendon Remodeling

ET is any structured, planned, and repetitive physical activity with the final objective of improving physical fitness [56]. ET is the most common method to apply mechanical loading to tendons [57]. During mechanical loading, ECM supplies tensile strength to the tendon [58]. Tendon cells detect mechanical forces as stimuli that are transduced to biochemical signals, eliciting cellular responses. Mechanical loading results in changes in cytoskeletal components, ECM organization, and gene transcription [30,59] The mechanotransduction mechanism is mediated by growth factors, receptors, intracellular pathways, and transcription factors [30,59]. Mechanotransduction is generally compound into three stages: mechanocoupling (physical load), cell–cell communication and the effector response. The communication throughout a tissue to distribute the loading message and the response at the cellular level to affect the response [60]. Cell responses to the ECM are determined by intrinsic properties that include adhesive affinity, matrix stiffness, fiber alignment, and matrix density [61]. In these terms, integrin-mediated adhesions between cells and ECM are essential for cell function. Cell–matrix adhesions have been described as mechanosensitive since they regulate biochemical signaling. One of the key functions of cell–matrix adhesions is to detect, transmit, and respond to mechanical signals [62]. Accordingly, tendon represents a dynamic, mechanoresponsive tissue [60]. Evidence indicates the existence of a threshold, or set-point at the applied strain magnitude, at which the transduction of the mechanical stimulus seems to impact the tensional homeostasis of the tendons [63]. Furthermore, mechanical forces are involved in type I collagen protein synthesis and ECM components in animal and human tendons [64,65,66,67]. In this sense, increased type I collagen formation was observed after acute exercise (2 and 72 h post-training) in the peritendinous tissue of runners [68], suggesting an adaptation to acute physical loading. Accordingly, 4- and 11-weeks post-training, increased turnover of collagen type I was observed in a peritendinous CT region. Interestingly, the authors observed that synthesis and degradation processes elevated after 4 weeks of training, whereas the anabolism was maintained after 11 weeks, generating a net synthesis of type I collagen in the tendon tissue [53]. Collagen synthesis increased in the patellar tendon as a result of a single bout of acute exercise, and this effect was maintained 3 days later [69]. On the other hand, different stress patterns result in different cellular reactions, which depend on the strength of applied stress. For example, repetitive tension applied during one day stimulated proliferation and apoptosis in contrast to extended stress periods [70], which supports the fact of ECM reacts differently depending on the nature and duration of the exercise [71].

Long-term effects of ET were observed on structural and mechanical properties of swine tendons [72], indicating that mechanical forces play a fundamental role in training-induced tendon adaptations. In this sense, striking adaptations in tendons in response to training were documented in a recent systematic review, although the authors highlighted the evident variability between and within studies. Despite the dose-response or time-course of tendon adaptation in response to the first months of training being controversial, larger tendon CSA was associated with long-term (years) training without evidence of differences in material properties [73]. In fact, mechanical loading (and ET) seems to induce both structural and functional adaptations in tendons, including collagen organization, CSA, tendon thickness, elastic energy, force, and stress-strain characteristics (Young’s modulus) [63,74,75,76].

In addition to upregulation of collagen content, treadmill running elevated the expression of mechanical growth factors (MGF) and enhanced the proliferative potential of TSPCs in both the patellar and CT of mice [77]. On the other hand, excessive mechanical loading caused significant differentiation of TSPCs into non-tendon cells [77]. Aberrant mechanical stimulation also favors the production of MMPs, growth factors, and prostaglandins, which can all induce defects in ECM remodeling and, consequently, the induction and progression of tendinosis [78,79]. Mechanical stretching also upregulated MMP gene expression, resulting in elevated interstitial amounts of MMP-2 and MMP-9 in human peritendinous tissue [80]. In fact, pro-MMP-2 levels elevated 3 days after an exercise bout in the peritendinous tissue of young men [81]. On the other hand, Arnoczky and colleagues (2004) observed that ex vivo static tensile loading inhibited the upregulation of MMP-1 expression induced by load deprivation in tendon cells [82]. Moreover, MMP-1 expression in tendon cells can be modulated by different amplitudes and frequencies of cyclic tensile strain [83]. Cyclical load also induced the release of degraded cartilage oligomeric matrix protein (COMP), a non-collagenous ECM protein [58]. In fact, we demonstrated that RT upregulated proteins responsible for ECM organization of tendons, including COL1A1, as well as a series of proteins associated with metabolic/development processes, such as FABPH, GELS, S100A6, TRFE, and serum albumin (ALBU) [84]. Although there is a tendency of upregulation in COMP levels in tendons of young rats submitted to RT, no statistical difference was reported. On the other hand, RT restores the aging-induced downregulation of COMP levels [84].

Further adaptations in tendon tissue have been observed in response to RT, including changes in tissue thickness, strength, resistance to damage, blood flow, and normalization of the fibrillar morphology [75,80,85], although plyometric training did not change force, stiffness, elastic energy, strain, or modulus in CT [76]. RT (or strength training) consists of an ET model that includes concentric and eccentric muscle actions against loads (workload) with the objective of achieving a specific training outcome [86]. RT is the most effective method for developing musculoskeletal strength and has been widely prescribed by the major health organizations [87,88,89]. In view of divergences concerning studies using experimental models, our research group sought to investigate the effects of RT on the biomechanical properties of aged rat tendons. Moreover, mechanical loading placed on tendon (and muscle) tissue induces collagen expression by the mechanotransduction mechanism, with involvement of TGF-β, CTGF, and IGF-I [90,91,92]. Together with skeletal muscle responses, we observed that RT significantly elevated the gene expression of key growth factors (TGF-β and CTGF) in gastrocnemius (GAS) and soleus (SOL) muscles of old rats [93].

Whereas tenocytes extracted from flexor and extensor tendons behave similarly when exposed to mechanical strain in vitro, distinct regions of human CT (insertion site, enthesis, and mid-tendon) have been shown to respond differently to mechanical loading. For example, some tendon regions have a high amount of glycosaminoglycans (GAGs), ensuring the ability of the tissue to support strong compressive forces [94].

In our studies on the effects of AAS and aging, we showed that RT improved the biomechanical responses in the CT of rats [95]. RT seems to improve the capacity of tendon tissue to support more stress, although whether different regions of tendons are affected by mechanical loading (and other factors) is still under debate.

In view of tendon repair, the influence of mechanical loading is not the same for different tendon types and tendon regions [96]. The differences in the outcomes probably arise from variation in mechanical loads placed on different tendon areas (for example, mid-tendon versus enthesis) and also on different tendons [30]. In this regard, we recently reported that the CT, SFT, and DFT of trained rats reach values of maximum extension [95]. Furthermore, the CT and SFT of old trained (OT) rats were capable of withstanding more stress, while the DFT showed greater resistance to maximum strain. Maximum extension and strain were observed in the CT of trained groups (either old or young animals) when compared with their sedentary counterparts [95]. Moreover, the CT of young trained rats demonstrated higher capacity to support stress when compared with OT animals, supporting our previous findings regarding the deleterious effects of aging on tendon adaptations. RT also blunted the age-associated low energy absorption in the CT and elevated energy absorption in the SFT.

RT ameliorated the age-induced low energy absorption of the CT and SFT [95]. OT rats presented an increase in elastic modulus (tendon stiffness) of the SFT in relation to young trained animals. RT also promoted greater capacity for producing tendon strength (maximum load) and resisting applied tension (maximum stress) in the SFT of old animals. Moreover, the SFT showed greater capacity to absorb energy to failure and less displacement to maximum load in OT rats when compared with the young trained group. However, RT had no effect on tendon CSA, whereas DFT stiffness was reduced in trained rats. Taken together, we suggest that RT may be considered an effective component that mitigates the age-induced detrimental effects on tendon adaptations [84,95].

In view of transcriptional regulation induced by mechanical loading, we observed that RT induced upregulation of genes linked to ECM homeostasis of tendons, including COL-I, COL-III, CTGF, TGF-β1, IGF-Ia, VEGF, MMP-2, TIMP-1, Bgn, Fmod, tenascin C, and decorin. Whereas RT mitigated the age-associated decrease in IGF-Ia and MMP-2, a dramatic increase in gene expression of CTGF and VEGF were observed in OT animals [97]. In addition, RT promoted higher blood vessel volume density and increased peritendinous sheath cells in young animals, as well as volume density of tendon proper cells being elevated in the proximal region of the CT [97]. Thus, we suggest that RT exerts a protective effect against age-induced tendon adaptations, including the adverse remodeling of ECM proteins and a reduction in the volume density of blood vessels as well.

Proteoglycan content was also elevated in the CT of trained young rats [97], suggesting enhanced elasticity of the CT in response to RT. Moreover, RT prevented age-induced calcification and induced an increase in proteoglycan content in the CT [97]. Furthermore, crimp arrangement of the CT was more intense in response to RT. This was observed quantitatively; however, no significant differences were observed in the birefringence microscopy. Taken together, our studies indicate that RT creates a suitable environment to restore tendons from damage [97].

Considering both collagen synthesis and gene expression analyses, a physiological increase in mechanical loading seems to be beneficial for tendons, whereas a decrease in mechanical loading is detrimental for tendon formation during tissue development [30]. However, RT did not affect the mechanical properties or dimension of the patellar tendon of old individuals [98], even though elevated ECM remodeling has been observed in response to RT [84]. Thus, our results suggest that an RT model might be an effective intervention against aging-induced deleterious effects on the biomechanical and morphological properties of tendons, supporting the use of RT as an important strategy to trigger beneficial adaptations in tendons. Our data are further supported by studies using running training on a treadmill [4,99].

## 4. Effect of AAS on Tendon Remodeling

A plethora of studies regarding the abuse of AAS have been conducted in subjects (athletes and casual fitness enthusiasts) owing to the potential risk factors for public health [11,12,13,14]. In fact, these drugs induce substantial effects on the morphology of skeletal muscle and development of strength [9,100,101]. Furthermore, high doses of AAS have been shown to affect the collagen metabolism [102], which could lead to the supposition that AAS may affect both the mechanical and morphological properties of tendons. These observations led to studies investigating the effects of AAS on tendons, particularly the effects of high doses of AAS on tendon injuries. In the late 1980s and early 1990s, studies investigated the effects of high doses of AAS, concomitant with ET on the structure of tendons [103]. The authors observed collagen dysplasia and reduced volume fraction in tendons of the flexor digitorium longus muscle of mice submitted to ET concomitant with the use of AAS, however, of note, no differences were observed in trained or AAS-treated animals. Changes in the diameter of collagen fibrils were also observed in AAS-treated mice, submitted or not to ET [103,104], although biochemical testing revealed no alterations in fibril diameter, type-III collagen, or fibronectin expressions in another study [105]. In conclusion, the author suggests that AAS-induced collagen abnormalities depend on the duration of AAS treatment and might evoke clinical disorders and tendon rupture [106,107]. Subsequently, morphometric analyses of rat tendons were studied in response to AAS and ET. In combination or not with ET, AAS produced augmented crimp angle and reduced crimp length, which has implications for tendon mechanical properties and functional behavior [108,109]. Tendon elasticity was reduced in rats that received AAS. Finally, the authors suggested that the combination of AAS and ET might predispose tendons to injury and rupture, which has been supported by other studies with humans [110,111]. Biomechanical tests showed that AAS induced a stiffer tendon that presented less elongation, as well as impaired energy absorption, elongation, and toe-limit elongation [105,112]. Interestingly, the effects were reversible following discontinuation of the use of AAS [105]. Publications on the topic of ultrastructural analysis of tendons date from the early 20th century. Considering that tendon ruptures could be associated with tissue fibrosis, investigators have focused on the effects of AAS and ET on collagen metabolism. In this sense, high doses of AAS enhance collagen synthesis in soft connective tissues (muscle, bone, and tendon) [102].

Considering that progress has been made since then on molecular approaches, our research group sought to investigate the effects of AAS and RT, which reflects a mechanical loading condition on tendon homeostasis, particularly events related to ECM remodeling – as will be discussed later in this review. Firstly, the Marqueti study demonstrated that AAS administration (Deca-Durabolin and Durateston) impaired the training-induced increases in MMP activity of CT of rats [113]. It should be noted that a marked elevation in metalloproteinase 2 (MMP-2) activity was observed only in trained rats, suggesting that mechanical loading is a potential stimulus for tendon turnover (synthesis, degradation, and re-synthesis). Skeletal muscle seems to be more sensitive than tendons in response to mechanical loading [90], which is supported by studies demonstrating elevated MMP expression in the muscle [93,114,115,116,117]. On the other hand, MMP expression seems to be differently regulated by mechanical loading in distinct regions of tendon (proximal and distal) [16], while, MMPs response might also be dependent on type and frequency of stimuli [118,119,120,121]. In this sense, further research is needed to a better understanding of mechanical loading effects on MMP expression in the tendons. Interestingly, a pronounced increase in serum corticosterone levels was observed in trained rats that received high doses of AAS [113]. Considering that AAS might inhibit collagen synthesis as well as which corticosterone treatment suppresses the synthesis of collagen types I and III, the study showed evidence of the detrimental effects of AAS on collagen synthesis by decreasing the MMP-2 proteolytic pathway.

In view of our preliminary results, we were interested in investigating whether MMP-2 activity would be altered in different regions of distinct tendons, since tendon composition and ECM elements vary according to regions (proximal and distal) [122], stage of development, mechanical loading, and aging [15]. Thus, MMP-2 activity was measured in the proximal and distal regions of 3 tendons—CT, superficial (SFT), and deep flexor tendon (DFT)—in response to AAS supplementation and RT [16]. The study showed that the SFT responded more to RT, in association with recruitment of the flexor digitorum superficialis muscle, while DFT was more related to the movement of distal parts of the digits. SFT, in turn, responded more to AAS compared to DFT and CT. However, the disparity in responses among the tendons might be due to the distinct metabolism and loading demands imposed on tendon regions during RT.

Considering our previous findings related to collagen metabolism, we hypothesized that molecular events would affect functional properties of rat tendons. To date, no investigations have been carried out regarding the effects of AAS supplementation and ET. On this basis, Marqueti and colleagues (2011) compared the biomechanical properties in the CT, SFT, and DFT of rats treated with high doses of AAS and submitted to RT [123]. The authors observed that the CT accommodated less energy and resisted tensional load more than the SFT and DFT in the sedentary group. SFT was slightly affected by RT, AAS, or a combination of both. On the other hand, increased elastic modulus of the SFT was observed in trained rats supplemented with AAS in comparison with the sedentary group. The DFT, in turn, supported more stress in response to RT, while AAS alone demonstrated no effect. Furthermore, the DFT showed a reduction in the displacement at maximum load when training and AAS were associated. In other words, the AAS reversed the effect of exercise and induced the DFT to exhibit less deformation. The tendons of AAS-treated rats submitted or not to RT exhibited either a decreased capacity to resist tension (i.e., decreased maximum strain) or accommodate levels of tensile strength (i.e., decreased toe region) together with reduced deformability (i.e., increased elastic modulus). In this sense, elevated stiffness, stress and modulus of patellar tendon were observed in AAS users, suggesting higher risk of tendon injury [124]. Accordingly, the different responses between the tendons are supported by biomechanical analysis in a study using an RT model (jump training), where the authors outlined the movement of rats during training [123]. In fact, AAS supplementation revoked the RT-induced effects on the CT. The study emphasizes that the loss of tendon flexibility might raise the risk of tendon rupture during training in individuals who abusively use AAS.

According to our findings regarding biomechanical properties, AAS supplementation and RT might regulate gene expression of key elements responsible for ECM homeostasis. In this regard, mRNA levels of type I collagen-α1 (COL1A1), type III collagen-α1(COL3A1), tissue inhibitor of metalloproteinase 1 (TIMP-1), tissue inhibitor of metalloproteinase 2 (TIMP-2), MMP-2, (insulin-like grow factor I-Ea) IGF-IEa, (glyceraldehyde-3-Phosphatase Dehydrogenase) GAPDH, (connective tissue growth factor) CTGF, and (transforming growth factor beta 1) TGFβ-1 were evaluated in different regions of the CT, SFT, and DFT of rats [125]. RT did not alter COL1A1, COL3A1, MMP-2, or IGF-IEa mRNA levels in the tendons (except for the distal region of the DFT), which is partially in accordance with other studies using different ET models (voluntary wheel running or squat apparatus) [126], as gene expression of type I collagen (COL-I), type III collagen, (COL-III), and TGFβ-1 expression was upregulated in the CT of trained female rats [90]. On the other hand, ET has been shown to increase collagen turnover, including some degree of collagen synthesis, in human tendons [80]. AAS supplementation, in turn, decreased the RT-induced upregulation of IGF-1 mRNA levels in the intermediate region of the SFT. AAS supplementation reduced the expression of COL1A1 in proximal and distal regions of the CT as well as in the proximal region of the DFT. In the SFT and DFT, AAS combined or not with RT reduced the expression of COL1A1 in both intermediate and distal regions. Similarly, AAS treatment reduced COL3A1 expression in both the distal region of the CT and intermediate region of the DFT. AAS combined or not with RT decreased COL3A1 expression in both intermediate and distal regions of the SFT as well as in the proximal region of the DFT. AAS also reduced MMP-2 expression in the proximal and distal regions of the CT and in the proximal region of the DFT, while AAS combined with RT decreased MMP-2 expression in the distal region of the CT and intermediate region of the DFT. The lower MMP-2 activity observed in different regions of tendons [16], provides further information concerning the harmful effects of AAS on tendons at a transcriptional level [125]. Accordingly, AAS enhanced TIMP-1 mRNA levels in the proximal region of the CT, while AAS combined or not with RT reduced TIMP-1 expression in the intermediate region of the SFT [125]. These findings might support the decreased gene expression and activity of MMP-2 in tendon tissue, as TIMP-1 has been demonstrated to play a pivotal role as an endogenous antagonist of MMPs [127].

As the potential effects of AAS and RT were observed in different tendons (and regions) [125], it is conceivable that the ultrastructure of tendons could be affected. Thus, qualitative analyses were carried out on the CT, SFT, and DFT of rats regarding volume density (Vv%) of the adipose cells, blood vessels (blood vessel lumen, endothelial cells, and perivascular sheath), peritendinous sheath cells, and tendon proper cells (fibroblasts and fibrochondrocyte-like cells) [128]. The major finding of this study relies on the distinct morphological adaptations in response to RT, which was linked to their composition and regional function. For example, different arrangements were found in the intermediate region of the SFT and DFT from the sedentary group, as well as increased cellularity and blood vessels. In addition, the SFT and DFT were similar in respect to material properties, which is consistent with displacement at the maximum load, stress, strain, and elastic modulus [123]. On the other hand, the CT accommodated less energy and resisted the tensional load more promptly than the SFT and DFT in the sedentary group. These findings might suggest that different tendons perform distinct functions in a set of movements by modulating the remodeling of the ECM proteins and adapting differently to new physiological demands [128]. RT altered the tendon proper cells and peritendinous sheath, which affects cells (adipocytes, synovial-like cells, fibroblasts, and fibrochondrocytes) and tendon vascularization (blood vessels) [128]. The Vv% of the tendon proper cells in the SFT and DFT (proximal and distal regions) and cell Vv% of the peritendinous sheath of the SFT (all regions) and DFT (distal region) increased significantly in response to RT. These findings indicate increased metabolism in tendons, which was related to greater blood vessel Vv% and adaptation following tissue remodeling during 7 weeks of training. In contrast, AAS combined with RT seems to mitigate the RT-induced increase in blood vessel Vv% in the CT (proximal region), SFT (intermediated region), and DFT (all regions). This combination (AAS and RT) also induced higher accumulation of adipose cells in the proximal region of the CT. Adipose cells play an important role with respect to the facilitation of movement between fascicles of tendons and dissipation of stress and tension at the attachment sites. Synovial-like cells were observed around the peritendinous sheath of trained rats (SFT, proximal, and intermediate region), AAS-treated rats (CT, distal region), and rats treated with AAS and submitted to RT (intermediate region of SFT, and proximal and intermediate regions of DFT). Synovial cells produce synovial fluid, which reduces tissue friction at the myotendinous interface [25]. The presence of synovial-like cells in the intermediate region of the SFT in trained rats supplemented with AAS suggests the possibility of an injury site. In fact, we have previously reported that AAS and training combined induced stiffer tendons [123], which might lead to the occurrence of tendon injuries.

RT elevated collagen content, as measured by hydroxyproline concentration, in the distal region of the CT, and intermediate and distal regions of the DFT. Curiously, high levels of collagen content were observed in sedentary animals, particularly in regions containing more cells. One feasible explanation for high levels of collagen content might be related to the paw position of rats in their cages [128]. AAS supplementation combined or not with RT reduced collagen content in some tendon regions, which might indicate adverse effects of AAS abuse on collagen metabolism of tendons. Collectively, the negative effects of AAS supplementation observed in this study include a reduction in blood vessels, increased adipose cell Vv%, the presence of synovial-like cells, and a reduction in collagen content [128]. Thus, we demonstrated compelling evidence regarding the effects of abusive AAS supplementation on tendon homeostasis of rats. Taken together, our findings suggest that AAS, particularly when combined with high-intensity RT, exerts harmful effects on tendon tissue in terms of ECM remodeling, and molecular and biomechanical adaptations. In view of these findings, RT might play important role in various aspects of tendon remodeling. On the other hand, a recent systematic review highlights the need for future research on this topic [129].

## 5. Effect of Aging on Tendon Remodeling

In the early 1980s, a study revealed that aging induced morphological and biochemical alterations in rabbit tendons, including an increase in ECM proteins and collagen concentration and a decrease in water content. Structural alterations in elastic fibers and a decrease in cell numbers of tendons were also observed in response to aging [2]. It should be noted that the drop in cell number and reduced synthetic activity of tendons seem to be attributed to the maturation process rather than the aging phenomenon *per se* [5]. On the other hand, while diameter increases with advancing age, the thickness of fibers is affected in a distinct pattern [2]. It is also becoming evident that age-induced alterations in tendon structure might influence mechanical function [5]. Mechanically, aging appears to be associated with a reduction in the modulus and strength of tendons [5], leading tendons to be more susceptible to injuries [130,131]. In this sense, the low metabolic rate of aged tendons slows the rate of recovery and healing processes after activity and injury, as the metabolism of aged tendons is more dependent on anaerobic energy pathways than aerobic pathways [24].

As the cell-to-matrix ratio gradually decreases with aging, morphological changes occur in the tendon cells. Furthermore, tenoblasts turn into tenocytes (and occasionally vice versa) and become very elongated while the nucleus-to-cytoplasm ratio increases. The cell processes are required to maintain strict contact between the cells and matrix components and then compensate the decreasing number of cells and increasing amount of tendon matrix [24]. Moreover, Gagliano and colleagues (2018) demonstrated the ability of tenocytes to maintain ECM remodeling in aged tendons, supporting the hypothesis that structure and biomechanical properties are preserved with advancing age [132]. He collagen synthesis of tendons reduces drastically with advancing age [24], even though the diameter of collagen fibrils appears to remain unchanged [133]. The relative distribution of collagen fibril sizes may change with aging, while total content of collagen fibrils (volume fraction) remains unaltered [5,133,134]. Furthermore, studies reveal that tendons become more susceptible to weakening [58] and stiffer with advancing age [135,136], although when the effects of aging are separated from those of the maturation process, a decrease in tendon stiffness has been observed [137,138]. In fact, gastrocnemius tendon stiffness was lower in older adults compared to young individuals [139]. The author suggested that more compliant tendons of the elderly allow the muscle fibers to shorten more. In addition, we have observed increases in connective tissue content in the soleus and gastrocnemius of old rats [93], even though no biomechanical analysis was performed. However, our results are in accordance with the Reeves study, suggesting lower stiffness of the gastrocnemius tendon, and more likelihood of injury. Indeed, mechanical properties of tendons are impaired with aging [139].

Substantial alterations in cross sectional area (CSA) and material properties (maximum load, stress, strain, load, elastic modulus, energy at failure, and displacement at maximum load) were observed in the CT, superficial (SFT), and deep (DFT) flexor tendon of older rats, suggesting that aging leads to increased tendon stiffness [95]. Moreover, aging induced low energy absorption in the CT and SFT. Overall, we demonstrated that aging reduced the ability of the CT and SFT to absorb energy, while decreased extension and increased elastic modulus were observed in the DFT of old rats, indicating an important effect of aging in different patterns of tendons.

In experimental models, the normal aging process is characterized by a series of morphological and biochemical alterations in tendons, including decreased collagen turnover [19], increased elastin levels [140], accumulation of partially degraded collagen within the matrix [141], changes in age-related cross-links as a result of glycation reactions [142], and decreased levels of abundant non-collagenous protein, as observed by the lower levels of cartilage oligomeric matrix protein (COMP) [143]. The age of animals used in experimental models varies between species. In this sense, rats ranging in age from 18 to 30 months are usually used in studies addressing this matter [144,145,146]. Accordingly, we demonstrated that aging caused meaningful reductions in proteins related to ECM organization, including COMP and collagen alpha-2(I) chain (COL1A2) as well as proteins associated with metabolic/development processes, such as carbonic anhydrase (CAH3), fatty acid-binding protein heart (FABPH), (acid-binding protein-4) FABP4, parvalbumin alpha (PRVA), gelsolin (GELS), protein S-100A6 (S100A6), and serotransferrin (TRFE) [84].

Despite recent advances regarding the effects of aging on collagen concentration, compelling evidence has indicated that aging alters collagen cross-linking (both enzymatic and non-enzymatic reactions) rather than collagen concentration in tendons in experimental models on aging [134]. Those non-enzymatic glycation/glycosylation reactions result in production of advanced glycation end products (AGE) in tendon tissue [147]. AGE accumulation is dependent on collagen turnover rates [148] and accumulates to a higher extent in tendon than in skeletal muscle as these tissues show low and high turnover, respectively [149,150]. Glycation increases the distance between collagen molecules within tendon collagen fibrils and, therefore, affects their molecular structure [151]. AGEs also contribute to the loss of water in aged tendons [2], since cross-links lead to dehydration of collagen [152]. The cross-links are important to stabilize the collagen fiber and thereby contribute to the mechanical properties and stiffness of the tendon [142,153]. Collagen concentration was reduced in the patellar tendon of elderly men, even though collagen cross-linking was elevated. This might be considered as a crucial mechanism to maintain the mechanical properties of tendons with advancing age [154]. Aging has been shown to be a potential factor that leads to AGE accumulation [155], resulting in stiffer and more load-resistant tendons [156,157]. Furthermore, recent studies demonstrated a reduction in tendon stiffness with aging [158,159,160]. While there is no consensus regarding the collagen content in aged tendon, the most remarkable structural alteration is an increase in AGE cross-links, which might lead to an increase in tendon modulus and strength [5]. In vitro observations indicate that AGE favors increased tensile stress and stiffness with aging [161,162].

Effects of aging on tendon mechanical properties (modulus and strength) are still a matter of intense research. For example, stronger and stiffer tendons have been observed with aging [133,163,164], while other studies have shown weaker and more compliant tendons [165,166,167,168]. Furthermore, aging seems to affect the viscoelastic properties of tendons [169,170]. Relevant age-dependent differences in ultimate stress, relaxation rate, and percent relaxation were observed in the tail tendon of old rats, where relaxation rate and percent relaxation decreased with age [167]. As mechanical properties of the tendon influence the muscle-tendon unit function [5], it is worth investigating the underlying events of aging-associated tendon development, particularly ECM remodeling. Indeed, dramatic changes in the rate of force development, elastic energy return, and electromechanical delay [17,171,172] might affect balance and mobility in elderly people [160].

As relevant alterations have been observed in terms of functional properties in different aged tendons, we speculate that ECM proteins might be modulated at a molecular level. In fact, we demonstrated that key genes associated with ECM remodeling were downregulated in the tendons of old rats [97]. Our study revealed that COL-I content and GAGS were reduced in the CT of older sedentary (OS) animals, while biglican (Bgn) and fibromodulin (Fmod) content were not altered with aging. Gene expression of COL-I, COL-III, IGF-Ia, MMP-2, TIMP-2, and Bgn were inhibited in aged tendons. MMP-2 activity of tendons was not affected in response to aging. OS animals exhibited higher adipose cell volume density in the proximal compared to distal region of the CT. Aging also reduced blood vessel volume density in both regions (proximal and distal) [97]. Furthermore, OS animals presented substantial calcification in the distal region of the CT [97]. In this regard, reduced levels of Bgn, GAGS, and Fmod seem to regulate ectopic ossification (calcification) in the CT [21,71]. Thus, we speculate that reduction in GAGS content might contribute to CT calcification of old rats [97], therefore, favoring greater CT stiffness in OS animals, which is in accordance with our previous findings [95]. While calcification increases with aging, mechanical loading induced a marked reduction in CT calcification in old mice submitted to ET [99], suggesting that mechanical loading plays a fundamental role in age-related tendon calcification.

In addition to the transcriptional approaches, we carried out proteomic analysis of the CT in old rats. We demonstrated high levels of fatty FABP4 with aging [84]. Thus, the higher adipose cell volume density observed in tendons of old sedentary rats [97] might support, at least in part, our previous findings regarding the FABP4 protein [84]. Considering that FABP4 is a protein associated with obesity and metabolic syndrome [173], we suggest aging-induced accumulation of adipose tissue in tendons, as confirmed by morphologic analysis [97]. In this context, aging has been associated with a lower rate of cell proliferation and a reduction in the number of TSPCs of tendons [5,174,175,176], while ET can mitigate age-induced deleterious effects on TSPCs proliferation [177], suggesting the importance of ET on tendon adaptations [4,5,77].

## 6. Conclusions

**Figure 2:**
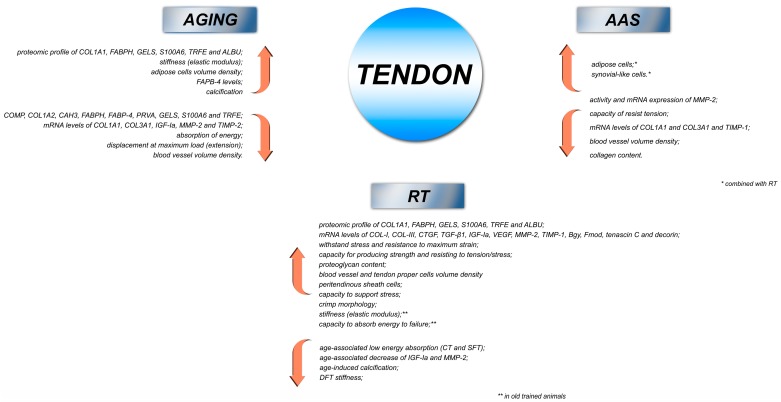
Integrative model based on our findings, indicating the major effects of AAS, RT, and aging on tendons of rats. AAS—anabolic androgenic steroids; RT—resistance training; COL-I—type I collagen; COL-III—type III collagen; COL1A1—type I collagen-α1; COL3A1—type III collagen-α1; TIMP-1—tissue inhibitor of metalloproteinase 1; TIMP-2—tissue inhibitor of metalloproteinase 2; MMP-2—metalloproteinase 2; IGF-IEa—insulin-like growth factor I-Ea; CTGF—connective tissue growth factor; TGFβ-1—transforming growth factor beta 1; VEGF—vascular endothelial growth factor; Bgn—biglycan; Fmod—fibromodulin; COMP—cartilage oligomeric matrix protein; FABPH—acid-binding protein heart; FABP4—acid-binding protein-4; GELS—gelsolin; S100A6—protein S-100A6; TRFE—serotransferrin; ALBU—serum albumin; CAH3—carbonic anhydrase; PRVA—parvalbumin alpha.

Our results suggest that AAS, particularly when combined with high-intensity RT, exerts harmful effects on ECM remodeling, and molecular and biomechanical adaptations in rat tendons. Moreover, we provide further information regarding the harmful effects of AAS on tendons at a transcriptional level. We also demonstrated the beneficial effects of RT against the age-induced tendon adaptations of rats. Therefore, our studies might contribute in terms of clinical approaches in favor of the benefits of ET against tendinopathy conditions and provide a warning on the harmful effects of AAS misuse on tendon development. Our results are summarized in Figure 2.

## Abbreviations

AAS	anabolic androgenic steroids
ALBU	serum albumin
Bgn	biglycan
CAH3	carbonic anhydrase
COL1A1	type I collagen-α1
COL3A1	type III collagen-α1
COL-I	type I collagen
COL-III	type III collagen
COMP	cartilage oligomeric matrix protein
CSA	cross-sectional area
CT	calcaneal tendon
CTGF	connective tissue growth factor
DFT	deep flexor tendon
ECM	extracellular matrix
FABP4	acid-binding protein-4
FABPH	acid-binding protein heart
Fmod	fibromodulin
GAGS	glycosaminoglycans
GAPDH	glyceraldehyde-3-Phosphatase Dehydrogenase
GELS	gelsolin
IGF-IEa	insulin-like grow factor I-Ea
MMP-2	metalloproteinase 2
PRVA	parvalbumin alpha
RT	resistance training
S100A6	protein S-100A6
SFT	superficial flexor tendon
TGFβ-1	transforming growth factor beta 1
TIMP-1	tissue inhibitor of metalloproteinase 1
TIMP-2	tissue inhibitor of metalloproteinase 2
TRFE	serotransferrin
TSPC	tendon-derived stem/progenitor cells
VEGF	vascular endothelial growth factor
